# Heavy metal transformation in mangrove ecosystems: a multi-scale perspective from intertidal dynamics to plant adaptive responses

**DOI:** 10.48130/forres-0026-0001

**Published:** 2026-01-29

**Authors:** Yaseen Khan, Bing Bing Ye, Adnan Anwar Khan, Ling-Feng Miao, Fan Yang

**Affiliations:** 1 Center for Eco-Environment Restoration Engineering of Hainan Province; School of Ecology, Hainan University, Haikou 570228, China; 2 School of Breeding and Multiplication (Sanya Institute of Breeding and Multiplication), Hainan University, Sanya 572025, China; 3 School of Tropical Agriculture and Forestry, Hainan University, Haikou 570228, China

**Keywords:** Mangroves, Heavy metal transformation, Phytoremediation, Intertidal dynamics, Rhizosphere, Plant adaptation

## Abstract

Mangrove ecosystems function as vital biogeochemical interfaces between terrestrial and marine environments, playing a crucial role in transforming heavy metals (HMs). However, this ecosystem is heavily impacted by climate change and anthropogenic activity, including an increase in HM toxicity. The current review synthesizes understanding of HM transformation across three interconnected levels: tidal dynamics, rhizosphere processes, and plant adaptation strategies. Initially, tidal inundation affects the distribution, speciation, and mobility of HMs by altering sediment biogeochemical properties, including pH, redox potential, salinity, and microbial activity. Further, tidal effects influence metal immobilization and remobilization, thereby impacting HM behavior within the rhizosphere, which serves as a secondary barrier to metal transport. Activities in the rhizosphere, including the presence of microbes, generate redox micro-gradients, and release organic ligands that facilitate metal complexation, precipitation, and detoxification. The synergistic interactions between roots and microbes support rhizoremediation in mangrove systems, lowering HM toxicity, and enhancing sediment stability. Additionally, mangroves employ various structural, physiological, and biochemical strategies, including selective metal uptake, excretion, internal detoxification systems, and the activation of antioxidant enzymes, to reduce HMs-induced stress. However, adaptation mechanisms differ among species and are influenced by interactions between tidal regimes, rhizosphere conditions, and plant traits. Integrating the three hierarchical levels—tide, root, and plant—highlights that mangrove ecosystems function as self-regulating biogeochemical systems capable of stabilizing and transforming HMs under dynamic environmental conditions. Such integrative mechanisms advance nature-based remediation strategies and reinforce mangroves' role as effective natural barriers against HM pollution, thereby contributing to sustainable coastal management and ecosystem resilience in a changing global environment.

## Introduction

Mangrove ecosystems are coastal wetlands dominated by woody plants that thrive in brackish and intertidal marine environments. These unique ecosystems occur along tropical, subtropical, and warm-temperate coastlines, acting as vital natural buffers against coastal flooding, storm surges, and erosion. Globally, mangroves are distributed across approximately 118 countries and territories, primarily between 25° N and 25° S, with an estimated total area of around 17 million hectares^[1]^. They support rich biodiversity, hosting about 60%–75% of the world's tropical coastal flora and fauna, and nearly 90% of marine organisms depend on mangrove habitats at some stage of their life cycle^[2]^. Moreover, mangroves provide crucial ecological and economic benefits, including wood resources, food supplies, carbon (C) sequestration, and nutrient cycling, thereby sustaining both marine life and coastal communities^[3]^. Asia holds the highest proportion of global mangrove coverage and biodiversity (41.9%), followed by Africa (20.1%), the Caribbean and Central America (13%), South America (11.1%), New Zealand and Australia (7.3%), Pacific Islands (4.5%), North America (1.8%), and Middle East (0.3%)^[4]^.

In China, mangroves cover approximately 22,000 hectares and are mainly distributed along the coastal mudflats of Hainan, Guangdong, Guangxi, Fujian, Zhejiang, Hong Kong, Macao, and Taiwan^[5]^. In China, 28 true mangrove species and 11 semi-mangrove species have been historically reported, supporting over 300 benthic animal species, 142 insect species, 96 phytoplankton species, 55 macroalgae species, 26 zooplankton species, seven reptile species, and 10 mammal species^[6]^. Subsequently, He et al.^[7]^ documented 854 organism species associated with Chinese mangrove wetlands, including 136 fungi, 13 actinobacteria, seven bacteria, and 441 microalgal species. More recently, Hu et al.^[8]^ reported that 26 true mangrove species are currently recognized in China, and noted the presence of newly introduced species, such as *Laguncularia racemosa* and *Sonneratia apetala*, which now occur within some mangrove ecosystems. Occupying the dynamic interface between land and sea, mangroves experience periodic tidal inundation, salinity fluctuations, and sediment deposition^[9]^, all of which shape their structure, function, and ecological productivity.

**Figure 1:**
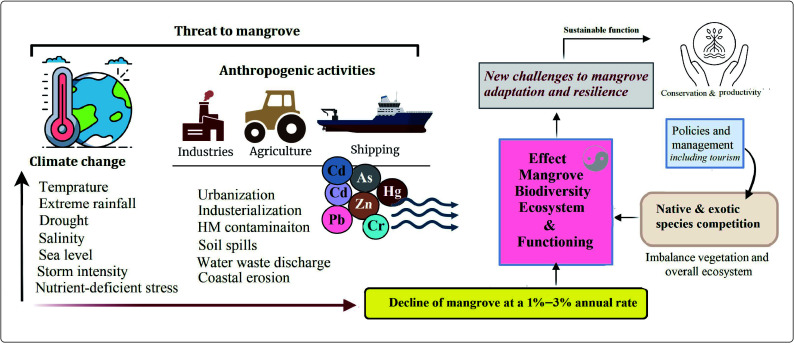
Increasing climate change and anthropogenic pressures negatively affect mangrove ecosystem functioning by reducing community stability and increasing heavy metals (HMs) toxicity. These impacts are further compounded by outdated management policies that intensify competition for resources between exotic and native species, destabilizing biodiversity and vegetation structure. Consequently, mangroves face major challenges to their resilience and adaptive capacity under changing environmental conditions. Addressing these challenges and enhancing mangrove resilience will improve their functions and help conserve them for the future amidst increasing climate change.

Despite their ecological importance, mangroves are among the most threatened ecosystems globally. Between 1980 and 2005, approximately 3.6 million hectares of mangrove forests were lost due to urbanization, pollution, aquaculture, overexploitation, and agricultural expansion^[10]^. Over 90% of mangroves occur in developing countries, where annual depletion rates (1%–3%) are particularly high^[11]^. Similarly, mangrove coverage in China declined sharply from 48,000 ha in 1973 to 18,000 ha by 2000^[12]^. Nonetheless, climate change and human stressors pose a significant threat to mangrove ecosystems and their functions, including some policies related to the introduction of new species that create imbalances and pose challenges to adaptation and mangrove resilience (Fig. 1). Projections suggest that an additional 25% of global mangrove forests could disappear by 2025^[13]^. Such degradation not only reduces biodiversity but also increases the vulnerability of approximately 15 million people to coastal flooding.

Among various pollutants, heavy metals (HMs) represent one of the most serious threats to mangrove ecosystems due to their persistence, toxicity, and bioaccumulative potential. Rapid industrialization and urbanization have accelerated the release of HMs into coastal environments, primarily through agricultural runoff and industrial wastewater^[14]^. However, the transformation and fate of HMs in mangrove systems are regulated by complex interactions in the mangrove ecosystem, including tidal dynamics, rhizosphere processes, and plant adaptation mechanisms. The intertidal zone (subject to fluctuating hydrological and biogeochemical conditions) acts as a natural laboratory for metal transformation^[15]^. Gradients in inundation frequency, duration, and depth create variations in sediment properties and redox potential, which in turn influence metal mobility and speciation^[16,17]^. In addition to the tidal impact on metals, the mangrove rhizosphere plays a crucial role in processes such as accumulation, transformation, and detoxification through root exudation, redox regulation, and microbial mediation^[18]^. However, the processes of HMs immobilization and detoxification, mediated by tide levels and rhizosphere interactions, also depend on HMs toxicity, location, salinity, mangrove species, and their composition.

The adaptive mechanisms that have evolved in mangrove species to withstand HMs stress, salinity, and inundation play a crucial role in HMs detoxification, making them essential for both current and future ecosystem resilience. Nevertheless, differences among species in their capacities for metal uptake, accumulation, and detoxification are still poorly characterized. However, numerous studies have investigated metal concentrations and transformations^[19−21]^. The understanding of the integrated roles of intertidal dynamics, rhizosphere processes, and plant adaptations in mediating HMs transformations remains overlooked. Therefore, this review provides an updated overview of HMs transformations in mangrove ecosystems, emphasizing the roles of intertidal dynamics, rhizosphere processes, and plant adaptive strategies. By integrating recent studies, it elucidates the biogeochemical mechanisms operating within mangrove sediments and rhizospheres, as well as external and internal plant tolerance mechanisms. Furthermore, it aims to refine predictive frameworks for ecological risk assessment and to support the conservation and sustainable management of mangrove ecosystems under increasing pressures from anthropogenic and climatic changes.

## HM contamination in mangrove ecosystems

From a plant physiological perspective, HMs are broadly categorized into essential and non-essential elements. Essential metals such as copper (Cu^2^^+^) and zinc (Zn^2^^+^) are required in trace amounts to sustain various physiological and biochemical processes. However, when their concentrations exceed critical thresholds, they exert toxic effects similar to those of non-essential metals, such as lead (Pb), arsenic (As), and cadmium (Cd)^[22]^. Excessive accumulation disrupts membrane transport systems, disrupts nutrient uptake, and inhibits plant growth and metabolism^[23]^. Plants like mangroves and their ecosystems are recognized as natural sinks for various HMs, including chromium (Cr), mercury (Hg), Cd, Pb, Zn, Cu, As, and nickel (Ni), which pose significant ecological toxicity^[24,25]^. Some metal ions, such as Pb^2^^+^, Cd^2^^+^, Hg^2^^+^, Fe^2^^+^, Zn^2^^+^, and Cu^2^^+^, can negatively impact the growth and productivity of particular mangrove species^[26−28]^. Consequently, mangrove sediments often act as major repositories for these HMs.

Over recent decades, anthropogenic pressures, such as deforestation, salt extraction, urbanization, industrial discharge, aquaculture expansion, domestic sewage release, and agricultural runoff, have substantially increased HM loading in coastal regions^[29,30]^. Earlier assessments indicated that approximately 29,720 km^2^ of China's offshore zones, including mangrove habitats, were heavily contaminated^[31]^. More recent studies, particularly in southern China and Hainan Island, have reported that Cu, As, and Hg are the dominant pollutants contributing to elevated ecological and human health risks, with Cu showing the highest contamination levels, Cd categorized as considerable, Hg as moderate, and other metals as low^[32,33]^. Despite these elevated pressures, mangrove forests mitigate the impact by acting as the first biogeochemical barrier between terrestrial and marine environments. However, the toxic metal ions continue to impair plant growth, affect physiological and molecular processes, and also disrupt the soil microbial community, which is essential for organic matter decomposition^[34−36]^.

The ecological capacity of mangroves to sequester, transform, and immobilize HMs has been weakened by poor management policies, including the introduction of exotic species a few decades ago, along with a net decline of approximately 9.3% in mangrove area due to tourism development and other anthropogenic disturbances^[37,38]^. A notable case observed in Hainan is the introduction of the exotic species *S. apetala* from Bangladesh in 1985, which has altered the composition of the native species vegetation and mangrove ecosystem. Due to pronounced differences in their ecophysiological characteristics, the introduced *S. apetala* has become dominant over the native *Bruguiera sexangula*, which grows more slowly but exhibits greater salinity tolerance^[39,40]^. Consequently, changes in vegetation structure and species dominance can significantly influence the dynamics of HMs, highlighting the importance of further investigation into the interactions between native and exotic mangrove species in Hainan to support more effective management and restoration strategies. Beyond vegetation factors, mangrove ecosystems and their function are also regulated by hydrological and biogeochemical processes, including freshwater inflow, tidal flushing, microbial activity, and rhizosphere interactions^[41]^. Disruptions to these processes may promote the remobilization and migration of metals through sediments and water, heightening the risk of bioaccumulation within aquatic food webs. Such transfers pose a serious threat to the health of marine organisms, including fish and crustaceans, and ultimately to humans^[42−44]^. Thus, maintaining a functionally and sustainably balanced mangrove ecosystem is crucial not only for mitigating HMs contamination, but also for preserving key ecological services such as shoreline stabilization, C sequestration, and fisheries productivity. The continued degradation of mangrove ecosystems poses a serious threat to the ecological integrity, environmental resilience, and economic value of coastal and marine ecosystems worldwide.

### Sources, concentration, and distribution of HMs

Most HMs in mangrove ecosystems are attributed to anthropogenic activities, including wastewater discharge, industrial operations, agricultural runoff, urbanization, and marine traffic. Previous reviews by Kulkarni et al.^[27]^ and Silva et al.^[45]^ highlighted the release of HM from agricultural activities, domestic sewage, mining, and industry into the mangrove ecosystem. However, specific metals can often be traced to distinct sources: Hg, Mn, and Fe primarily originate from industrial discharges such as papermaking and printing; Pb, Ni, Cr, Zn, and Cu are mainly derived from industrial emissions, maritime transport, and traffic-related pollution; whereas Cd is primarily linked to agricultural activities involving fertilizers and livestock manure^[28,31,46,47]^. A smaller fraction of metals, such as Zn, Ni, and Co, may also arise from natural sources, including geological weathering. For instance, volcanic activity and geochemical cycling contribute less frequently, but they can act as significant sources of HM influx in certain regions^[48]^. HMs' concentration in mangroves and their major sources across various regions worldwide are varied, as shown in Table 1.

**Table 1:** Concentrations of HMs in mangrove sediments worldwide and their primary sources. Concentrations correspond to total HMs in mangrove surface sediments.

Heavy metal	Concentration (mg·kg^−1^)	Location/country	Major sources	Ref.
As	14.0	Xiamen Bay, China	Industrial wastes, shipping activities	[49]
	3.6−18.3	Bay of Bengal, India	Tidal waters, fresh water rivers, and storm water runoff	[50]
	0.52–35	Sydney, Australia	Urbanization and population growth	[51]
Pb	20.07	Zhanjiang Bay, China	Agricultural production activities	[52]
	7.38	Meghna River Estuary, Bangladesh	Anthropogenic sources, particularly near shipbreaking	[53]
	105	Shenzhen, China	Rapid urbanization and industrialization	[54]
	0.7–13.37	São Paulo State, Brazil	Fishing and waste disposal	[55]
	42.27	Sanya-Hainan, China	Wastewater's discharge	[56]
Cu	18.24	Zhanjiang Bay, China	Rapid urbanization and industrialization	[52]
	35.74	Meghna River Estuary, Bangladesh	industrialization	[53]
	400	Shenzhen, China	Anthropogenic sources, particularly near shipbreaking	[54]
	12.44	Sanya-Hainan, China	Wastewater's discharge	[56]
	0.74–9.42	São Paulo, Brazil	Fishing and waste disposal	[55]
	256.0–356.6	Farasan Island, Saudi Arabia	Sewage runoff, farming practices, and industrial discharge	[20]
Cd	0.09	Sanya-Hainan, China	Home and industrial waste waters discharge	[72]
	1.04	Farasan Island, Saudi Arabia	Sewage runoff and industrial discharge	[27]
	0.25−0.42	São Paulo State, Brazil	Fishing and waste disposal	[71]
Zn	52.76	Sanya-Hainan, China	Discharge of waste and home usage discharge	[72]
	352	Shenzhen, China	Rapid urbanization and industrialization	[36]
	62.32	Meghna River Estuary, Bangladesh	Anthropogenic sources, particularly near shipbreaking	[70]
	29.5–36.8	Farasan Island, Saudi Arabia	Sewage runoff, farming practices, and industrial discharge	[27]
	4.49−49.51	São Paulo, Brazil	Anthropogenic activities, such as fishing and waste disposal	[71]

Variation in HM concentrations in mangroves reflects differences in local pollution sources, hydrology, and geochemical processes. For instance, the study conducted by Ahmed et al.^[57]^ reported that the concentrations of HMs in mud crabs, horseshoe crabs, and gastropods from the Sundarbans mangrove forest on the southwest coast of Bangladesh followed the order Fe > Zn > Pb > Cu > Cd. Similarly, in mangrove crabs (*Sesarma mederi*) from the upper Gulf of Thailand, accumulated metals in the order Cd > Cu > Pb > Zn^[58]^. In northern Vietnam, HM concentrations in mangroves follow the order Zn > Pb > Cr > Cu > As > Cd^[59]^. In the Rufiji Delta mangroves in Tanzania, metals are distributed as Cr > Zn > Ni > Cu > Pb > Cd^[60]^. In the Dongzhai Harbor mangrove wetland in Hainan, the pattern observed is Cr > Zn > Ni > Pb > Cu > As > Cd, with Cr and Zn as the dominant elements^[61]^. Along the Bay of Bengal (Southeast Asian countries), sediments showed that the concentration order was Cu > Zn > Mn > Cr > Pb > Co > As > Ni^[62]^. The distribution of HMs in mangrove sediments is greatly affected by tide levels because they influence the speciation, mobility, and transformation of these metals^[63,64]^.

**Figure 2:**
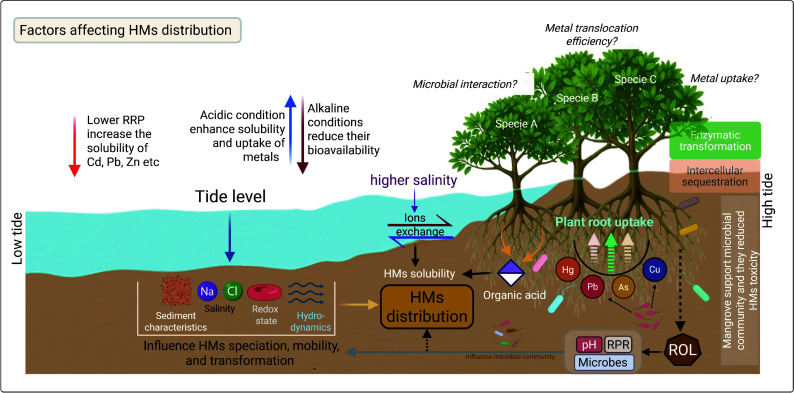
An overview of heavy metals (HMs) distribution and the key factors influencing HM dynamics and interactions within the mangrove ecosystem. Tidal level affects sediment characteristics, salinity, redox state, and overall hydrodynamics, which in turn regulate HM mobility and speciation, thereby controlling their distribution across tidal zones. Elevated salinity promotes ion exchange processes that alter HM solubility and accumulation in sediments. Additionally, organic acids released by mangrove roots enhance HM solubility, further influencing their spatial distribution. Microbial activity in the rhizosphere plays a crucial role in HM transformation, interacting with root-mediated radial oxygen loss (ROL) and root redox potential (RRP), which collectively influence sediment pH and HM bioavailability. The uptake, translocation (root-to-shoot ratio), and detoxification capacity of mangrove species further determine overall HM distribution. These processes depend on species-specific traits, including enzymatic transformation efficiency and intracellular sequestration mechanisms.

Tidal elevation gradients exert a substantial impact on sediment characteristics. For instance, tidal cycles play a pivotal role in regulating the redox state, salinity, and hydrodynamics of intertidal sediments—all of which influence the mobilization or stabilization of HMs and consequently alter their distribution^[65,66]^. During low tides, sediments are exposed to air, creating oxidative conditions that can release metals previously trapped as sulfide complexes^[67,68]^. Conversely, high tides bring in saline water, altering the ion exchange equilibria and affecting metal solubility and mobility^[69,70]^. These cyclic tidal processes create a dynamic environment in which HMs are continuously redistributed among solid-phase sediments, pore water, and the overlying water column. Besides tidal levels, the distribution of HMs in sediments is influenced by several other factors such as sediment physicochemical properties, microbial activities, rhizosphere processes, root exudation, and plant species (Fig. 2). These findings underscore the role of mangrove sediments as primary sinks for HMs, where concentration profiles fluctuate with local environmental conditions, pollution sources, and sedimentary processes.

### Common HMs and their toxicity

Recent studies have identified As, Pb, and Cu as the dominant HMs originating from anthropogenic inputs along mangrove coasts—primarily from sources such as antifouling Cu in shipping, legacy Pb from batteries and mining-derived As^[71−73]^. These metals are consistently detected in both sediments and mangrove tissues, providing robust evidence of their ecological significance. The accumulation and capacity of HM-tolerance, along with their detoxification mechanisms in dominant mangrove species are detailed in Table 2. Moreover, As, Pb, and Cu collectively represent the major stressors for mangroves and their molecular defense systems. Therefore, the following sections of this review focus on these three metals, and examine their toxicity in mangrove ecosystems.

**Table 2:** Comparative heavy metal accumulation, capacity, tolerance, and their detoxification mechanisms of the dominant mangrove species.

Mangrove species	Main metals accumulation	Accumulation capacity	Tolerance mechanisms	Ref.
*A. marina*	Pb, Cu, and As	Higher roots accumulate high levels of Pb and Cu; strong tolerance to As.	Root Fe-plaque formation, secretion of low-molecular-weight organic acids, antioxidant enzyme activation, restricted metal translocation to shoots	[18,74−77]
*K. obovata*	Pb and Cu	Higher Cu enrichment ~70%; strong Pb tolerance	Phytochelatin (PC-SH) synthesis, root sequestration, and cell wall binding	[74,76,78]
*K. candel*	Cu	Higher enrichment exceeds approximately 96% of its accumulated Cu	By increasing antioxidant activities, including SOD, POD, and CAT activities	[79]
*B. gymnorhiza*	Cu and Pb	Moderate accumulation without sustained tolerance at high exposure	Phytochelatin synthesis, antioxidant response, and limited ROL under Cu stress	[80,81]
*R. stylosa*	Pb and Cu	Moderate tolerates Cu up to 400 mg·kg^−1^	Restricted Cu translocation, decreased root permeability, root elongation, and ROL	[47,81]
*R. apiculata*	Pb	Moderate lower Pb accumulation	Root sequestration and exclusion	[82]
*A. corniculatum*	As	High As tolerance but low overall HM accumulation	Root immobilization and exclusion, limited translocation	[51,83]
*A. alba*	Pb	Moderate to high leaf accumulation	Higher Pb accumulation and translocation of HMs to leaves	[84]
*A. ilicifolius*	Pb	Moderate to higher accumulation		[71]
*S. hainanensis*	Cu	Lower Cu accumulation	Lower tolerance mechanism against Cu	[85]

#### As toxicity

As is a non-essential and highly phytotoxic metalloid, it ranks among the most serious inorganic contaminants in natural waters globally. It originates from both natural processes, such as geological weathering and volcanic activity, and anthropogenic sources, including fossil fuel combustion, mining, and the use of arsenic-based agrochemicals^[86,87]^. In China, its primary inputs are derived from industrial (47.19%), agricultural (33.13%), and traffic-related (13.03%) activities^[88]^. Elevated As concentrations have been reported globally, including 14.0 mg·kg^−1^ in Xiamen Bay, China^[49]^, 3.6–18.3 mg·kg^−1^ in the Sundarbans mangrove forest, India^[50]^, up to 70 mg·kg^−1^ in Espírito Santo, Brazil^[89]^, and 0.52–35 mg·kg^−1^ in Sydney Estuary, Australia^[51]^. However, the bioavailability of As is strongly influenced by several environmental factors. For instance, fluctuations in pH can promote As desorption into the overlying water by disrupting chemical bonds, thereby reducing its retention in sediments^[90]^. Likewise, the transformation of insoluble arsenic compounds into soluble ionic forms can increase As mobility^[91]^. Moreover, microbial reduction of As(V) to the more mobile As(III) species can further alter their distribution and availability in aquatic systems^[92]^.

As exerts toxicity in mangrove plants primarily due to its chemical similarity to phosphate. Arsenate (As (V) interferes with cellular metabolism by reacting with thiol (-SH) groups in proteins and substituting for phosphate in metabolic pathways. It enters plants through phosphate transporters, inhibiting growth^[93]^ or is reduced to arsenite (As [III]), which induces the production of reactive oxygen species (ROS), leading to lipid peroxidation and cellular damage^[94]^. As toxicity typically manifests as inhibited root growth, altered membrane permeability, and disrupted water and nutrient uptake. Although mangrove species possess notable tolerance to As stress, the degree of toxicity, and the underlying mechanisms of resistance vary among species and tissues. *Aegiceras corniculatum* exhibits substantial As tolerance and severely affects its roots^[51,83]^. Similarly, *Avicennia marina* shows reduced As accumulation accompanied by enhanced secretion of low-molecular-weight organic acids (OA) in roots compared to shoots^[18]^. Overall, evidence indicated that As targets the roots and middle aerial parts of mangrove plants more effectively than shoots^[51,95]^.

Mangrove species exhibit diverse strategies to tolerate As stress. *Acanthus ilicifolius* mitigates As toxicity by enhancing photosynthetic efficiency, activating antioxidant defense systems, and accumulating osmo-protectants (e.g., proline and carotenoids) under high As exposure^[96]^. *A. corniculatum* alleviates As stress primarily through Phyto-stabilization and As exclusion, with most of the metal retained in the roots^[83]^. Meanwhile, *A. marina* reduces As toxicity by increasing the secretion of low-molecular-weight OA (citric, oxalic, and malic acids) and promoting the formation of Fe plaques in the rhizosphere, which influence As speciation and mobility^[18]^. Existing field studies provide limited insights into As accumulation and translocation due to fluctuating environmental concentrations and the absence of well-defined As gradients. Thus, controlled experiments incorporating a range of As concentrations and long-term monitoring are urgently needed.

#### Pb toxicity

Pb is one of the most pervasive pollutants originating from vehicle emissions, industrial discharges, mining, smelting, and petroleum refining^[97]^. These sources contribute to Pb deposition in coastal and marine sediments, including tropical and subtropical areas^[98]^. Pb interferes with the transport of organic compounds and disrupts the uptake of essential nutrients such as phosphorus (P), calcium (Ca), magnesium (Mg), and manganese (Mn), likely due to ionic competition, resulting in stunted plant growth^[99]^. It also inhibits enzyme activity by binding to thiol (-SH) and carboxyl (-COOH) groups or replacing cofactors in metalloenzymes, thereby impairing electron transport and photosynthetic processes^[100,101]^. Pb accumulation is mainly localized in roots, with limited translocation to aboveground tissues, resulting in reduced root function and suppressed seedling growth.

Mangrove species exhibit varied responses to Pb toxicity. For example, Huang et al.^[78]^ reported that phytochelatin (PC-SH) synthesis in *Kandelia obovata* and *Bruguiera gymnorhiza* plays a key role in mitigating Pb stress. Yan et al.^[102]^ observed that several cotyledonary mangrove species actively mobilize carbohydrates from leaves to roots under Pb exposure, increasing starch and malondialdehyde (MDA) levels while enhancing peroxidase activity to improve tolerance. Pb exposure can also elevate endogenous salicylic acid and jasmonic acid levels in seedling leaves, which are vital regulators of Pb stress resistance^[103]^. Among mangrove taxa, *A. marina* demonstrates relatively high Pb tolerance. Pb concentrations up to 800 mg·L^−1^ exerted minimal adverse effects on its seedling growth and emergence^[75]^. Similarly, *A. marina* roots accumulate higher Pb levels than *Rhizophora apiculata* and *S. alba*, indicating more effective Pb immobilization and detoxification, making it a promising species for Pb pollution mitigation^[82]^. Moreover, *L. racemosa* demonstrates notable Pb tolerance, and it is suggested that it has potential for remediation and restoration in contaminated mangrove areas^[104]^. Various factors affecting Pb uptake and distribution, such as salinity. In *R. apiculata* and *Avicennia alba*, Pb absorption patterns varied under salt stress—salinity significantly affected Pb content in stems of both species, while *A. alba* also showed higher Pb accumulation in leaves^[84]^. Similarly, mangrove species (e.g., *B. gymnorrhiza*, *K. obovata*, *R. stylosa*, *Aegiceras corniculatum*, *Acanthus ilicifolius*, *A. marina*) exhibited varied Pb tolerance under different salinity conditions^[71]^. These findings suggest that Pb distribution within plant organs is species-specific, and modulated by salinity. However, since Pb impairs essential metabolic processes and restricts plant growth, and considering its limited mobility to aerial parts, future research should focus on developing advanced phytoremediation strategies under realistic mangrove conditions.

#### Cu toxicity

Cu is a vital micronutrient, with 30% of its presence in chloroplasts, and it is crucial for various plant growth processes, though it is required in specific amounts. Cu deficiency causes serious nutritional problems in plants; however, when present in excess, Cu can become a significant environmental pollutant and a phytotoxic element. Industrial activities, mining, and the widespread use of Cu-based fungicides in agriculture, have led to the accumulation of Cu in soils and sediments^[105]^. Cu availability is strongly pH-dependent—its solubility increases under acidic conditions, which enhances its potential toxicity in the mangrove ecosystem^[106]^. Cu toxicity causes serious issues in plants, especially in mangroves. It induces oxidative stress, leading to structural damage, such as leaf deformation and impaired water transport^[107]^. Due to its redox activity, excess Cu generates reactive ROS that damage cellular components and disrupt metabolic functions, leading to decreased chlorophyll content and reduced photosynthetic rates, which ultimately suppress plant growth and productivity^[108−110]^. However, the level for Cu toxicity varies widely among plant species types and genotypes, reflecting inherent differences in Cu tolerance mechanisms^[111,112]^.

Mangrove plants have evolved complex mechanisms to detoxify excess Cu through exclusion, chelation, sequestration, and antioxidant defense. At the root level, Cu is immobilized through binding with lignin and suberin in cell walls and by iron plaque formation in the rhizosphere^[71,113]^. Within cells, Cu induces the biosynthesis of metallothioneins and phytochelatins, which chelate Cu ions and facilitate their sequestration into vacuoles via ABC transporters^[114,115]^. Cu homeostasis is further maintained by Cu chaperones that safely distribute Cu to target proteins. Additionally, Cu-induced oxidative stress is mitigated through enhanced activity of antioxidant enzymes such as SOD, CAT, and POD. The formation of radial oxygen loss (ROL) barriers in roots may also represent an adaptive strategy to limit Cu uptake and protect sensitive tissues^[81]^. However, variations in vegetation composition and climatic conditions influence mangrove responses, leading to species-specific differences in Cu tolerance and accumulation. These responses are further shaped by multiple interacting factors, including microbial activity, sediment properties, salinity levels, and tidal fluctuations, all of which contribute to the complexity of Cu dynamics in mangrove ecosystems.

Mangrove showed species-specific responses to Cu accumulation and tolerance. For example, *A. marina* and *K. obovata* exhibit substantial Cu accumulation, with enrichment rates of 74.2% and 70.5%, respectively^[74,76]^. However, in another study, *A. marina* seedlings showed growth inhibition only at > 120 µg·g^−1^ Cu^[77]^, while *B. gymnorhiza* and *R. stylosa* survived at 400 mg·kg^−1^ Cu in soil but with reduced root elongation^[81]^, suggesting that the accumulation capacity and toxicity depend on plant growth stage. Mangrove species, i.e, *K. candel*, were found to have up to 96% of their accumulated Cu confined to roots, accompanied by increased SOD, POD, and CAT activities, enabling seedlings to tolerate concentrations of up to 20 mg·L^−1[79]^. Furthermore, *R. stylosa* also shows substantial Cu uptake, whereas *S. hainanensis* accumulates lower Cu levels, indicating species-specific tolerance^[85]^. Conversely, Cu exposure significantly reduced growth and root permeability in *B. gymnorhiza* and *R. stylosa*, decreasing ROL and suggesting lower Cu tolerance^[80]^. Despite progress in understanding Cu toxicity and tolerance in mangroves, key knowledge gaps remain regarding species-specific toxicity thresholds and the molecular regulation of Cu transporters, sequestration mechanisms, and signaling pathways, which need to be addressed in future studies.

## Intertidal dynamics

The intertidal zone within mangrove ecosystems is particularly susceptible to contamination due to its exposure to fluctuating hydrological and biogeochemical conditions^[15]^. The unique hydrodynamic processes associated with tidal fluctuations significantly influence sediment–water interactions, redox dynamics, and the migration of metals within sediment columns^[116]^. Generally, higher concentrations of HMs are observed during high tide compared to low tide, indicating that tidal action regulates the movement of suspended sediments and the redistribution of metals across mangrove zones^[117,118]^. The impact of tidal on sedimentary processes is complex. During tidal fluctuations, shear forces at the sediment–water interface disrupt the oxidative surface layer, promoting the resuspension of particles. Under oxidizing conditions, soluble iron and manganese species transform into insoluble oxides, which possess high surface areas and strong adsorption capacities for HMs^[119]^. When hydrodynamic stress exceeds the cohesive forces among sediment particles, resuspension occurs, exposing the sediment to oxygenated conditions that alter pH and redox potential^[120]^. This leads to the release of adsorbed metals into the overlying water column and changes in the speciation of metals. Moreover, periodic inundation and exposure modify the equilibrium between interstitial water and surface sediments, influencing the diffusion and dispersion of metals.

During low tide, when sediments are exposed to air, the oxidation of organic matter and sulfides increases Eh, releasing associated metals. Simultaneously, iron and manganese ions form oxides that bind with HM, reducing their mobility^[121]^. Conversely, high-tide submersion decreases Eh, favoring the migration of metals from interstitial water to the overlying water. Salinity fluctuations associated with tidal processes further affect the solubility and speciation of metals, enhancing their mobility in estuarine systems^[122]^. Mangrove sediments, compared with adjacent mudflats, demonstrate markedly lower bioavailability of metals such as Cu(II), Zn(II), Cr(VI/III), Pb(II), and Ni(II)—by approximately 19%–79%. This reduction is most pronounced in mangrove zones, where high organic C content and root-associated processes promote immobilization of metals^[123]^. The dense mangrove canopy, accumulation of autochthonous organic matter, and active microbial communities collectively enhance sediment stability and contribute to the long-term sequestration of HMs, thereby reducing the ecological risks associated with contamination. Consequently, mangrove ecosystems function as effective natural barriers that mitigate HM exposure in intertidal environments. During sediment resuspension, suspended particulates serve as key carriers facilitating the transport and redistribution of HMs within the intertidal zone.

### Availability and distribution of HMs across tidal levels

The availability and distribution of HMs across tidal gradients are primarily influenced by sediment redox conditions, salinity, and organic content. Studies by Marchand et al.^[124]^, Botté et al.^[117]^, Reckhardt et al.^[125]^, and Silrat et al.^[126]^ consistently report higher metal concentrations during high tide compared to low tide. This pattern is attributed to the migration of metals through interstitial water under reduced conditions, followed by their precipitation as hydrous metal oxides upon exposure to oxygen. These oxides, being less soluble, become incorporated into sediment matrices, thereby explaining the elevated metal concentrations observed during high tide. At low tide, reduced and anoxic conditions promote the transformation of insoluble metal oxides into more soluble sulfide forms through sulfate reduction^[125,127]^. These redox-driven transformations facilitate the remobilization of metals into interstitial or overlying water. Furthermore, studies on sulfide minerals indicate that metal adsorption is predominantly controlled by surface hydroxyl interactions, which strongly influence metal behavior under anoxic conditions^[128]^. For instance, dissolved concentrations of Pb, Cd, Zn, Mn, and Cu have been observed to peak under low-salinity conditions^[129]^, reflecting the role of flocculation and colloidal iron oxyhydroxide dissolution in regulating metal distribution.

As shown in (Fig. 2), tidal processes affect sediment characteristics, salinity, redox state, and hydrodynamics, which in turn influence the availability of HMs in intertidal levels. However, studies have indicated various impacts from different angles within the mangrove ecosystem, including plant species accumulation, depth, organic acid release by plants, microbial processes, and organic matter decomposition. Studies have observed that significant bioaccumulation of metals such as Cu, Pb, Zn, Cd, Cr, and Hg in bivalves (*Crassostrea ariakensis*), fishes (*Mugil cephalus*), and crabs from coastal regions of China, India, and Indonesia^[130−133]^. Marchand et al.^[19]^ further demonstrated that variations in metal concentrations with depth are primarily controlled by diagenetic processes—particularly the cycling of iron and manganese associated with organic matter decomposition—rather than by direct anthropogenic inputs. Organic acid released by mangrove roots plays a crucial role in HM adsorption, immobilization, and distribution. Study by Hu et al.^[134]^ revealed that sediments with higher organic acid content exhibit a more substantial binding capacity for metals. In mangrove ecosystems, abundant litterfall, acidic conditions, and active microbial processes further enhance metal complexation and retention. Similarly, sediment acidity tends to increase with decreasing tidal level^[66]^, which accelerates organic matter decomposition and modifies metal mobility, thereby influencing the spatial distribution of metals within intertidal sediments. These findings suggest that fluctuations in tide levels effectively influence the chemical behavior and ecological effects of HMs in coastal sediments. The combined effect of redox-driven changes, organic matter processes, and microbial mediation influences both metal mobility and their accumulation in intertidal biota, with important ecological and environmental consequences.

### Intertidal influence on HMs mobility and transformation

The intertidal zone, situated between the high- and low-tide marks, is periodically submerged under tidal forces generated by gravitational interactions among the Earth, Moon, and Sun. These hydrodynamic processes strongly influence the fixation and mobility of HMs within sediments. Acting as major sinks for terrestrial and coastal pollutants, intertidal sediments are shaped by tidal amplitude, elevation, wave action, and wind energy. Collectively, these factors contribute to the high complexity and dynamism of material and energy fluxes within tidal environments^[135]^. The migration and transformation of HMs in intertidal environments are influenced by various factors, including hydrodynamic conditions, sediment properties, levels of dissolved oxygen, pH, salinity, and redox potential^[66]^. Periodic tidal flooding serves as a key abiotic driver in these ecosystems^[136]^, while environmental heterogeneity along elevation gradients further modulates these processes^[137,138]^. As tidal elevation increases, both the frequency of inundation and the severity of soil anoxia typically decrease. The dynamic alternation between submersion (marine-like) and exposure (terrestrial-like) phases in intertidal sediments induces pronounced fluctuations at the mud–air–water interface, accompanied by changes in physicochemical parameters such as dissolved oxygen and interfacial pressure^[139]^. Such tidal oscillations continuously reshape redox gradients and sediment chemistry, ultimately controlling the speciation, mobility, and bioavailability of HMs in intertidal systems.

Salinity, regulated by freshwater influx and tidal oscillations, plays a crucial role in controlling the solubility and mobility of HMs^[122]^. Elevated pH conditions promote the precipitation of free metal ions, whereas low pH enhances their mobility and bioavailability^[140]^. Studies conducted in the Hangzhou Bay region have demonstrated a positive correlation between HM concentrations and fine-grained sediment fractions, such as silt and clay^[141]^. Moreover, the depth and position of the anoxic interface fluctuate with tidal levels, further complicating metal mobility within the sediment column^[66]^. Upon entering marine systems, metal ions are typically adsorbed onto suspended particles that are subsequently transported landward by tidal action and deposited on tidal flats. In mangrove environments, dense root networks efficiently trap these particles, leading to the accumulation of both bound and free forms of HMs within sediments^[142]^.

Tidal cycles influence HM transformations through distinct mechanisms. During high tide, surface sediments become exposed to air, increasing redox potential and promoting the oxidation of organic matter and sulfides, which releases previously bound metals. Simultaneously, Fe and Mn ions oxidize to form hydroxides that can further adsorb and immobilize metals^[143]^. During low tides, the reduction in overlying water pressure facilitates the upward migration of porewater, enhancing solute exchange across the sediment–water interface. Conversely, during spring or high tides, intensified hydrodynamic activity disturbs the sediment surface, promoting the resuspension and redistribution of metals in ionic, organically bound, and particulate forms^[116]^. When shear stress exceeds a critical threshold, sediment resuspension exposes particles to oxidative conditions, thereby altering physicochemical parameters such as pH and EH, which in turn enhance metal remobilization and bioavailability^[144,145]^. Collectively, the interplay of redox fluctuations, hydrodynamic forces, and sediment–water exchanges under tidal influence drives the continuous remobilization and transformation of HMs, shaping their spatial distribution and persistence in coastal systems.

## Rhizosphere-mediated processes

The soil zone affected by mangrove roots plays a crucial role in controlling HMs, their detoxification, and their absorption by plants. The beneficial interaction between roots and sediments promotes accumulation and stability by collaborating with organic matter and clay. These elements help immobilize metals and act as natural biofilters through physical trapping, chemical changes, and biological absorption^[146]^. Additionally, root-released organic compounds, such as amino acids, sugars, and OA, serve as C sources for microorganisms and promote the production of degradative enzymes^[147]^. These enzymes can modify sediment pH, redox potential, and nutrient availability, thereby benefiting the microbial community and improving the mitigation of HMs toxicity^[148]^. In mangrove rhizospheres, bacteria (e.g., *Pseudomonas*, *Bacillus*, and *Rhizobium*) utilize enzymes such as oxygenases, dehydrogenases, and hydrolases to degrade complex pollutants, including polycyclic aromatic hydrocarbons and chlorinated compounds^[149]^. Additionally, the fungal hyphae extend throughout the sediment, enhancing the mangrove's root accessibility to pollutants and facilitating organic matter decomposition^[150]^. Similarly, ammonia-oxidizing archaea (e.g., *Nitrosopumilus* spp.) are found in the rhizosphere and support N cycling and pollutant transformation under oxygen-limited conditions^[151]^. These synergistic interactions between mangrove roots and their associated biotic and abiotic partners form the basis of rhizoremediation, which integrates microbial metabolism with root-mediated geochemical alterations to detoxify contaminants^[152]^. Through these coupled processes, rhizosphere-mediated interactions involve interactions from sediments to microbes, from HMs to nutrients, and from toxicity to plant detoxification, as reported by Seshadri et al.^[153]^ and Cipullo et al.^[154]^. In comparing mangrove rhizospheric and non-rhizospheric sediments. Furthermore, the rhizosphere-mediated mechanisms involved in HM detoxification in mangrove ecosystems are listed in Table 3. However, exploring rhizospheric microbiomes and their roles in pollutant degradation remains limited, and advancements in omics-based approaches—including metagenomics, transcriptomics, and metabolomics studies—are necessary to identify key genes, enzymes, and metabolic pathways involved in HM transformation and organic contaminant degradation. These developments will open promising avenues for creating targeted bioremediation strategies.

**Table 3:** Rhizosphere-mediated mechanisms contributing to heavy metal (HM) detoxification in mangrove ecosystems.

Mechanism type	Key processes/interactions	Representative agents (roots/microbes/compounds)	Effects on HMs	Ref.
Physical trapping	Sediment retention by complex root structures (prop roots, pneumatophores)	*Rhizophora*, *Avicennia*, *Ceriops*, and *Sonneratia* roots	Immobilization and reduced HM mobility	[155,156]
Chemical transformation	Redox alterations, pH modification, precipitation of metal oxides/hydroxides	Root oxygen leakage (ROL), organic acids, phenolics	Adsorption and precipitation of metals, and altered speciation	[153,157, 158]
Biological absorption and biosorption	Root and microbial uptake of metals	Mangrove roots with *Bacillus*, *Pseudomonas*, *Rhizobium*, *Aspergillus*, and *Penicillium*	Bioaccumulation and detoxification	[159,160]
Microbial enzymatic transformation	Redox reactions catalyzed by dehydrogenases, hydrolases, and oxygenases	Rhizospheric bacteria and fungi	Conversion to less toxic forms	[149,161]
Root exudate–microbe synergy	Release of amino acids, sugars, and organic acids promotes microbial metabolism	Root exudates; enzyme-producing microbes	Enhanced degradation of organic pollutants; improved HM tolerance	[148,149]
Mutualistic plant–microbe associations	Microbial assistance in nutrient cycling, stress tolerance, and HM detoxification	*S. apetala* rhizospheric consortia	Improved plant resilience and remediation efficiency	[162,163]

### Root-sediment interaction and sediment trapping

Root-sediment interactions influence HM mobility, shaping sediment chemistry and supporting microbial processes that sustain mangrove ecosystem functioning and restoration. Mangrove roots influence soil pH and nutrient availability, thereby influencing microbial community composition and enhancing the efficiency of pollutant degradation^[164]^. However, each of these processes strongly affects sediment characteristics and biogeochemical dynamics^[165,166]^. The intricate aerial root systems of mangroves, such as pneumatophores and prop roots, efficiently trap suspended sediments transported by tidal flows, which facilitate sediment deposition^[9]^. Although some studies suggest that mangroves enhance rather than initiate sedimentation, their structural complexity undoubtedly increases sediment retention and stability^[167−169]^. Sediments beneath mangroves serve not only as C sinks but also as natural archives of paleoenvironmental and sea-level fluctuations. These sediments are typically rich in organic matter and fine clays, which confer high cation exchange capacities that promote the strong binding of HMs^[170,171]^. As tidal waters flow through mangrove root networks, fine particles containing metals are trapped and immobilized within the sediment matrix, thereby reducing metal mobility and minimizing their transfer to adjacent aquatic systems. Moreover, oxygen leakage from mangrove roots creates localized oxidized microsites within anoxic sediments, altering redox potential and pH. These microscale gradients affect metal speciation and can induce the precipitation of metal oxides and hydroxides, which further adsorb HMs and reduce their bioavailability^[172,173]^.

Notably, mangrove roots trap a greater amount of sediment during low tide than at high tide, as they reduce tidal flow velocity and promote the deposition of suspended soil particles under calmer conditions. However, the efficiency of sediment trapping by mangroves is species-specific. Kathiresan^[174]^ reported that the *Avicennia*–*Rhizophora* interphase was more effective at trapping sediment than either the *Avicennia* or *Rhizophora* zones alone, retaining approximately 30%, 25%, and 20% more sediment, respectively, at low tide compared to high tide. Similarly, *Rhizophora* spp., which extend prop roots from the trunk and anchor within approximately 30 cm of sediment depth, capture more sediment than *Ceriops* spp.^[156]^. The magnitude of sedimentation is generally significant in trees forming complex root matrices, such as *Rhizophora* spp., and smallest in single trees like *Ceriops* spp.^[175]^. Other factors contributing to sediment accumulation and soil elevation include root length, longevity, and biomass turnover. The accumulation of long-lived roots through decomposition and compression of cellular material often increases soil volume, further promoting elevation gains^[176]^. Overall, mangrove roots function as effective sediment traps, and root–sediment interactions in mangrove ecosystems act as natural biofilters that integrate physical trapping, chemical transformation, and biological uptake to mitigate HMs pollution.

### HMs transformation in the rhizosphere

The rhizosphere is a narrow soil zone affected by root activity that plays a vital role in controlling HM mobility, transformation, and absorption in mangrove ecosystems. Within this microenvironment, metals can be mobilized, absorbed, accumulated, excluded, immobilized, or detoxified before reaching plant tissues^[177]^. Compared to bulk sediment, the rhizosphere exhibits distinct physicochemical and biological properties, including altered pH, redox potential, and microbial composition. These characteristics strongly influence metal speciation, bioavailability, mobility, and distribution^[178,179]^. OA secreted by mangrove roots can chelate or complex with HMs, enhancing their solubility, mobility, and subsequent uptake or detoxification within plants^[180]^. In contrast, phenolic compounds released from roots may bind metals to form less bioavailable complexes, thereby mitigating metal toxicity^[181]^.

Root exudates, microbial respiration (producing CO_2_), and redox-active processes can lower rhizosphere pH, which in turn influences enzyme activity (e.g., phosphatase activity, urease activity, and dehydrogenase activity) and microbial community composition. Acidic conditions generally enhance the solubility and uptake of certain metals, while alkaline conditions can decrease their bioavailability. Similarly, reducing (low redox potential) environments can increase the solubility and mobility of Cd, Pb, and Zn, reshaping their distribution within the rhizosphere^[153]^. Furthermore, enzymatic activities such as arylsulfatase, dehydrogenase, and urease serve as sensitive indicators of HM stress and are involved in facilitating metal transformations^[181,182]^. Another vital process is the ROL from mangrove roots into the surrounding sediments, which modifies rhizosphere redox conditions, pH, and the abundance of aerobic microorganisms^[157,183]^, thereby influencing HM speciation, precipitation, and mobility. Despite progress, it remains unclear how mangrove roots and rhizospheres respond to HMs contamination under changing environmental conditions, or whether these responses are consistent across species and habitats. Species-specific traits likely control metal tolerance and transformation, highlighting the need for further research to understand rhizosphere-mediated metal dynamics and sustain mangrove functions in contaminated coasts.

### Microbial mediation

Microbial remediation represents a sustainable biological approach for cleaning up contaminated environments. It primarily relies on microbial enzymatic catalysis and redox transformations, in which enzymes alter the oxidation state of metal ions, thereby facilitating their detoxification^[161]^. These microbially mediated redox reactions play a pivotal role in regulating sediment geochemistry, nutrient cycling, and overall ecosystem functioning. In mangrove ecosystems, such processes influence not only the fate of HMs and sediments, but also the bioavailability of nutrients essential for plant growth and ecosystem stability. Microorganisms—particularly rhizosphere bacteria—alleviate HM toxicity through various mechanisms, including intracellular sequestration, extracellular precipitation, adsorption, and enzymatic transformation^[181]^. Nevertheless, excessive HM accumulation can suppress microbial activity and inhibit key enzymatic processes^[184]^. Microbial biotransformation plays a crucial role in determining the bioavailability and mobility of metals in soil systems. The mobilization and immobilization of metal ions involve multiple enzyme-mediated mechanisms that are critical for detoxification^[185]^. During metabolic activities such as fermentation and respiration, microorganisms utilize pollutants as co-metabolic substrates, facilitating their breakdown and detoxification. The successful application of microbial bioremediation depends on selecting resistant microbial strains and elucidating their underlying mechanisms of metal resistance^[186]^. Contaminated sites often act as natural selection grounds for metal-tolerant microbial species. For biosorption-based remediation to be effective, the physicochemical characteristics, regeneration capacity, and stability of microbial biosorbents must be carefully evaluated^[187]^.

The study by Mallick et al.^[159]^ reported that microbes in the rhizosphere (e.g., *Bacillus*, *Pseudomonas*, *Penicillium*, and *Aspergillus*) exhibited substantial capacity to withstand HMs and transform them into less harmful forms, as later confirmed by Singh et al.^[160]^. Additionally, enzymatic detoxification allows microorganisms to convert HMs into less toxic forms, enhancing their potential for bioremediation applications. Microbial activity in the rhizosphere is essential for ecosystem recovery and the cleanup of contaminated soils and waters^[163]^. The interaction between plants and microorganisms underpin key phytoremediation mechanisms, including phytoextraction, phyto-transformation, phytovolatilization, and rhizoremediation. In mangrove ecosystems, species such as *S. apetala* form strong mutualistic associations with rhizospheric microbial communities^[162]^. These interactions enhance plant tolerance to environmental stressors, promote establishment, and contribute to HM detoxification through microbial mediation. Because plant roots serve as a habitat for beneficial microbes, species that host diverse and efficient microbial communities derive greater adaptive and physiological advantages. When plant–microbe interactions occur without detrimental effects on the host, mutualism is established. This mutualistic mediation enhances nutrient cycling, mitigates HM toxicity, and strengthens overall ecosystem resilience. However, the ecological functions of many microbial taxa and their interactions with plants and the rhizosphere remains insufficiently understood, particularly in environments where native and exotic species coexist and compete for adaptation and survival.

## Plant detoxification and adaptation strategies

Mangrove ecosystems are frequently exposed to multiple abiotic stresses such as salinity, anoxia, and HMs contamination. Both field and laboratory studies have demonstrated that mangrove systems are highly efficient at trapping metals and reducing their solubility and bioavailability^[188,189]^. However, excessive HM loading can exceed the sediment's binding capacity. Environmental disturbances such as prolonged dry periods or fluctuations in salinity may alter sediment chemistry, leading to the remobilization of previously bound metals^[190]^. Consequently, mangrove sediments can shift from functioning as metal sinks to acting as metal sources, often in association with anthropogenic disturbances^[173,191]^. Mangroves employ a range of physiological and biochemical mechanisms to detoxify HMs. These include metal chelation and sequestration within vacuoles, activation of antioxidant enzymes, accumulation of osmolytes, and symbiotic interactions with rhizospheric microbes that enhance HM immobilization and detoxification^[192,193]^. However, the efficiency of these mechanisms depends on plant species, habitat characteristics, and the genetic and physiological traits of individual mangroves.

Some mangrove species exhibit remarkable tolerance to HM exposure. For instance, *A. marina* seedlings showed no significant reduction in biomass during Pb exposure (up to 250 mg·L^−1^)^[102]^. Similarly, a minimal growth inhibition in *A. marina* under exposure to combined Pb, Cu, and Zn (Pb = 0–800 mg·L^−1^; Cu = 566 mg·L^−1^; Zn = 580 mg·L^−1^)^[75]^. *R. apiculata* exhibited no notable changes in root or leaf morphology after six months of Cr exposure (up to 500 mg·L^−1^)^[194]^. Furthermore, while *B. gymnorrhiza* and *R. stylosa* showed reduced root growth under high Cu exposure (up to 400 mg·kg^−1^), but both species still survived even at the highest Cu concentrations^[81]^. Conversely, several studies have reported adverse physiological effects of HMs on mangroves, including decreased chlorophyll content in *A. marina*^[195,196]^, *B. gymnorrhiza*, and *K. candel*^[197]^. Naidoo et al.^[77]^ observed a reduction of up to 60% in CO_2_ exchange rates under elevated exposure to Cu, Zn, and Pb, primarily due to decreased leaf conductance and chlorophyll degradation. HM stress can also alter the leaf C-nitrogen (N) ratio, as reported in *A. marina* exposed to Pb under controlled conditions^[102]^, and to multiple metals (Cr, Cd, Pb, Zn) in field environments^[198]^.

In addition, mangroves exhibit a suite of adaptive morphological and physiological traits that enable them to thrive in environments contaminated with metals, characterized by high salinity and anoxia. Recent observations of natural root grafting in *A. marina* populations suggest that interconnected root systems promote resource sharing and enhance resilience under stress^[199]^. Further structural adaptations, such as salt glands and thick, waxy leaves, help maintain ionic balance and reduce both salt and metal toxicity. Leaf succulence, observed in *Laguncularia racemosa*, *R. mucronata*, and *B. gymnorrhiza*, facilitates osmotic regulation during high HMs and salinity stress^[200,201]^. These characteristics may enhance mangrove functional traits under stressful conditions, and could positively influence environmental restoration efforts. These diverse and interconnected adaptive systems highlight the complexity of mangrove resilience.

### Variation in accumulation patterns

The accumulation patterns of HMs in mangrove plants are determined mainly by their concentrations in surrounding sediments, which serve as the primary metal source. However, both the capacity and preference for HM accumulation vary significantly among mangrove species and plant organs, reflecting differences in metal selectivity, physiological uptake mechanisms, and tolerance strategies. Species-specific variations are reported, with *S. apetala*, which shows the highest accumulation of Cu and As, while *K. obovata* exhibited the greatest Pb and Cd concentrations in Qi'ao Island, Zhuhai, China^[202]^. In the mangrove wetlands of Hainan, the adsorption capacity of different mangrove species for Cu, Zn, and Pb followed the order *Ceriops tagal* > *B. sexangula* > *K. obovata* > *A. corniculatum* > *A. marina*^[203]^. Another study conducted in the Futian mangrove reserve, Shenzhen, found that accumulation capacities for Cu, Pb, Zn, Ni, and Cr decreased in the order *S. caseolaris* > *S. apetala* > *K. candel*^[204]^. Differences are also evident among plant organs. In most mangrove species, fine roots tend to accumulate markedly higher concentrations of Cu, Pb, Zn, and Cr than branches, stems, or leaves^[204]^. Some species display unique metal partitioning patterns. For example, in *S. apetala* communities from the Daliao River watershed, Zn was found to be most concentrated in leaves, while Cu was primarily stored in perennial branches^[205]^. These findings highlight that a combination of environmental, physiological, and species-specific factors regulates metal accumulation in mangroves.

### External and internal detoxification mechanisms

To mitigate HMs contamination in intertidal sediments, several remediation strategies have been developed. Methods like physical remediation are primarily for HMs immobilization; however, this approach is suitable for treating moderate quantities of heavily polluted sediments and is typically applied over short distances in areas exposed to tidal influence^[206,207]^. Additionally, these methods can have a significant impact on mangrove ecosystems and intertidal natural processes. Mangrove species are particularly well suited for phytoremediation due to their rapid growth, high aboveground biomass, extensive root systems, and tolerance to high metal concentrations. They can efficiently translocate accumulated metals from roots to shoots, making them ideal for phytoextraction. For example, *B. sexangula* has demonstrated a high capacity to accumulate HMs while maintaining normal anatomical structures and physiological functions^[207,208]^. Cellular analyses have further revealed species-specific patterns of metal distribution within tissues^[197,209]^. The interaction between mangroves and HMs begins at the root–sediment interface, where the root cell wall acts as the first barrier, binding metal ions and preventing their entry into the protoplast. Additionally, mangrove roots develop lignified and suberized outer layers, as well as Casparian strips, which further restrict metal diffusion into internal tissues. During ROL, iron plaques often form on root surfaces, serving as adsorptive or physical barriers that control the movement of HMs. The efficiency of these plaques varies among species and environmental conditions^[210]^. Together, these external structures delay metal uptake, providing time for the activation of internal detoxification systems, such as chelation and antioxidant defenses. When external barriers are insufficient, mangroves activate intracellular detoxification mechanisms to maintain metal homeostasis. The plasma membrane plays a key role by regulating metal ion entry through selective transporters and efflux pumps^[42]^. Once metals enter the cytoplasm, mangroves synthesize a range of chelating and antioxidant compounds, including cysteine, glutathione (GSH), non-protein thiols (NPTs), phytochelatins (PCs), and metallothioneins, which bind HMs and neutralize their toxicity^[197,211]^.

**Figure 3:**
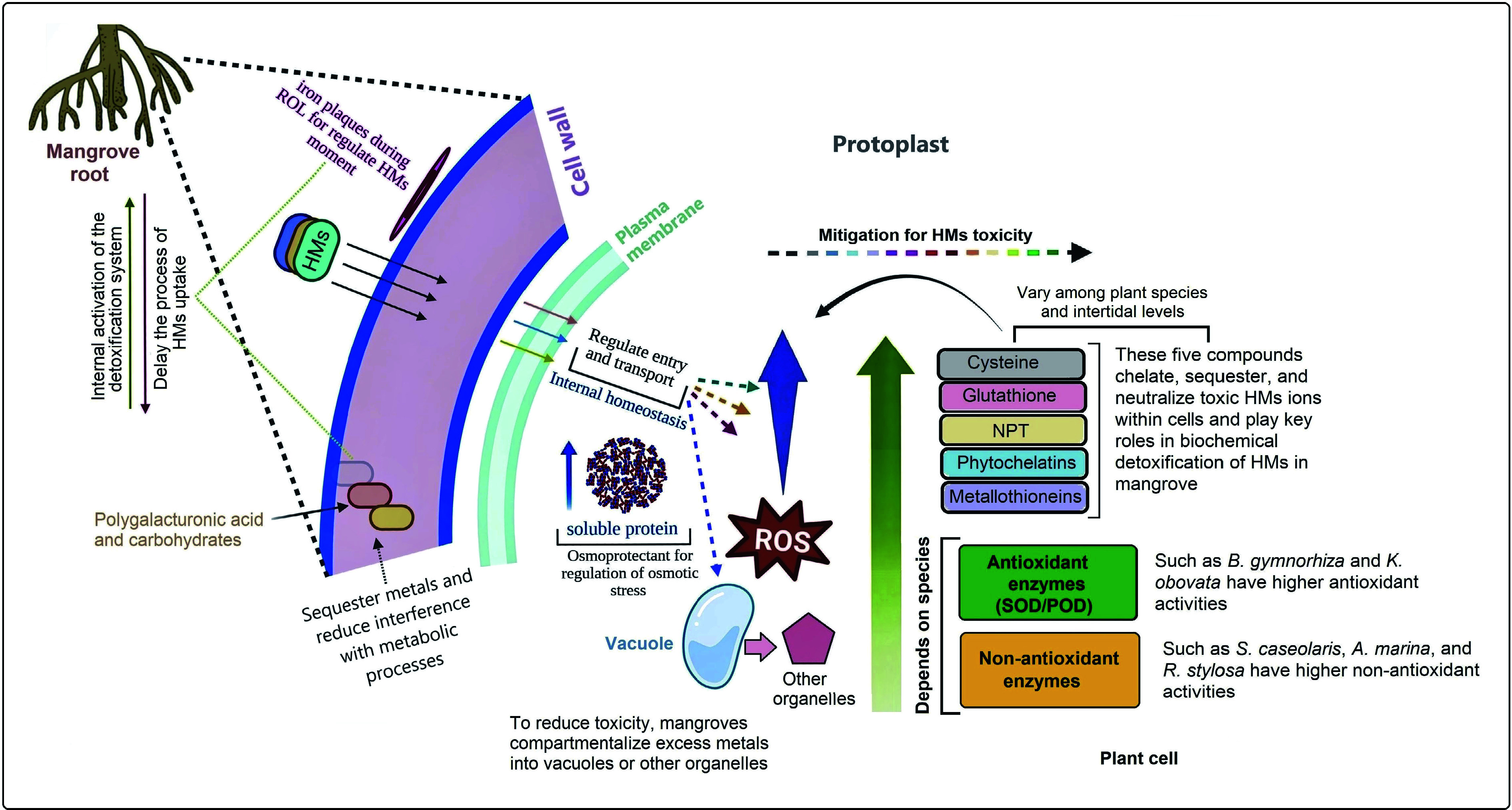
Cellular detoxification mechanisms of heavy metals (HMs) in mangroves. Mangroves absorb HMs through their cell walls and initially reduce toxicity by forming iron plaques during radial oxygen loss (ROL). This process helps maintain internal and external equilibrium between HM uptake and detoxification, facilitated by carbohydrates and polygalacturonic acids in the cell wall. The plasma membrane further regulates HM transport to preserve cellular homeostasis. To mitigate toxicity, mangroves enhance the synthesis of cysteine, glutathione, non-protein thiols (NPTs), phytochelatins, metallothioneins, and antioxidants, which scavenge reactive oxygen species (ROS) and neutralize HM ions. Additionally, osmo-protectants and soluble proteins are upregulated to alleviate osmotic stress. Under high HM exposure, cells compartmentalize metals into vacuoles and other organelles to limit cytoplasmic toxicity. Through these integrated physiological and biochemical processes, mangroves detoxify and tolerate HMs. However, the efficiency and specific mechanisms vary among species, reflecting differences in adaptive strategies to HM stress under contrasting tidal conditions.

HMs' exposure also induces the production of ROS, leading to oxidative stress and lipid peroxidation. To counter this, mangroves enhance both enzymatic antioxidant activities—such as superoxide dismutase (SOD), peroxidase (POD), and catalase (CAT)—and non-enzymatic antioxidants (e.g., phenolics, flavonoids, and ascorbate) (Fig. 3). To alleviate osmotic and oxidative stress, mangrove cells accumulate osmo-protectants, including soluble proteins, proline, and sugars, which regulate water balance and enhance stress tolerance. Excess metals are ultimately sequestered into vacuoles or organelles, forming stable complexes with proteins, polysaccharides, or OA. Electron microscopy studies have directly observed cadmium crystal deposits within vacuoles (including in other species), confirming their role in long-term metal sequestration^[101,212,213]^. Despite this multi-layered resilience, several aspects of mangrove biochemical detoxification remain poorly understood—particularly how native and exotic species adapt to increasing metal toxicity under climate change, vegetation decline, and interspecific competition in varying tidal zones. Also, future studies should focus on elucidating the epigenetic regulation mechanisms underlying HM tolerance in mangroves. This includes integrating single-cell and multi-omics analyses to reveal cellular-level detoxification pathways and exploring the functional role of rhizosphere microbiomes in enhancing both phytoremediation efficiency and overall ecosystem resilience.

### Integrated adaptive mechanisms

Through integrated structural, physiological, and molecular mechanisms, mangroves adapt to survive in saline, heavy-metal-contaminated, waterlogged, and anoxic environments. With specialized vegetative structures, mangrove root systems in anoxic sediments promote gas exchange and maintain ion balance^[214]^. Diverse root types such as prop roots (e.g., *R. mangle*), buttress roots (*Heritiera littoralis* and *Xylocarpus granatum*), stilt roots, knee roots, and cable roots (*Avicennia* spp., *S. caseolaris*) enhance both mechanical stability and aeration^[215]^. Roots also perform ultrafiltration at cortical membranes, selectively excluding HMs and sodium (Na^+^) and chloride (Cl^−^) ions to maintain ionic homeostasis^[216]^. As reported in *A. marina*, root grafting enables inter-root resource sharing, improving survival under HM and salt stress^[199]^. In addition to vegetative parts, mangrove stems also play crucial roles in both mechanical support and stress management. In several species, salts are actively excreted on stem and bark surfaces, thereby reducing the ionic burden within tissues^[217,218]^. Also, higher lignin and tannin content of mangrove stems provides resistance to decay, microbial attack, and mechanical damage from waves and storms. Moreover, mangrove leaves exhibit extensive specialization for desalination, HM detoxification, water conservation, and efficient photosynthesis under stressful environmental conditions. Many species possess salt glands (e.g., *Avicennia*, *Aegiceras*, *Acanthus*, and *Aegialitis*) that actively secrete excess salts^[219]^. Leaves also show succulence and thick cuticles to dilute internal salts and lower transpiration. These adaptations, including thickened leaves, have been observed in *R. mucronata* and *B. gymnorrhiza*^[201]^. Together, these structural adaptations across roots, stems, and leaves form an integrated defense system that ensures mangrove survival and functionality under adverse stress conditions.

Physiologically, mangroves sustain homeostasis through the coordinated regulation of redox balance, ion transport, and osmotic adjustment. Ion homeostasis is achieved via compartmentalization and selective transport of HMs and salt ions, often involving vacuolar sequestration. Vacuolar Na^+^/H^+^ exchangers (NHX), plasma membrane antiporters (SOS1), and H-ATPases collectively modulate cytosolic ion concentrations and pH^[220]^. Ferritin and calcium-signaling genes further stabilize ionic equilibrium by sequestering metal ions and regulating membrane transport^[221,222]^. ROS scavenging enzymes—such as SOD, catalase, and glutathione S-transferase (GST)—are upregulated under stress, mitigating oxidative damage and enhancing tolerance^[223]^. Osmotic homeostasis is further supported by the accumulation of compatible solutes such as proline, betaine, and soluble sugars^[224]^. Future research should focus on elucidating the intricate signaling networks and cross-talk among physiological pathways to clarify how mangroves coordinate multiple stress responses in changing coastal environments.

**Figure 4:**
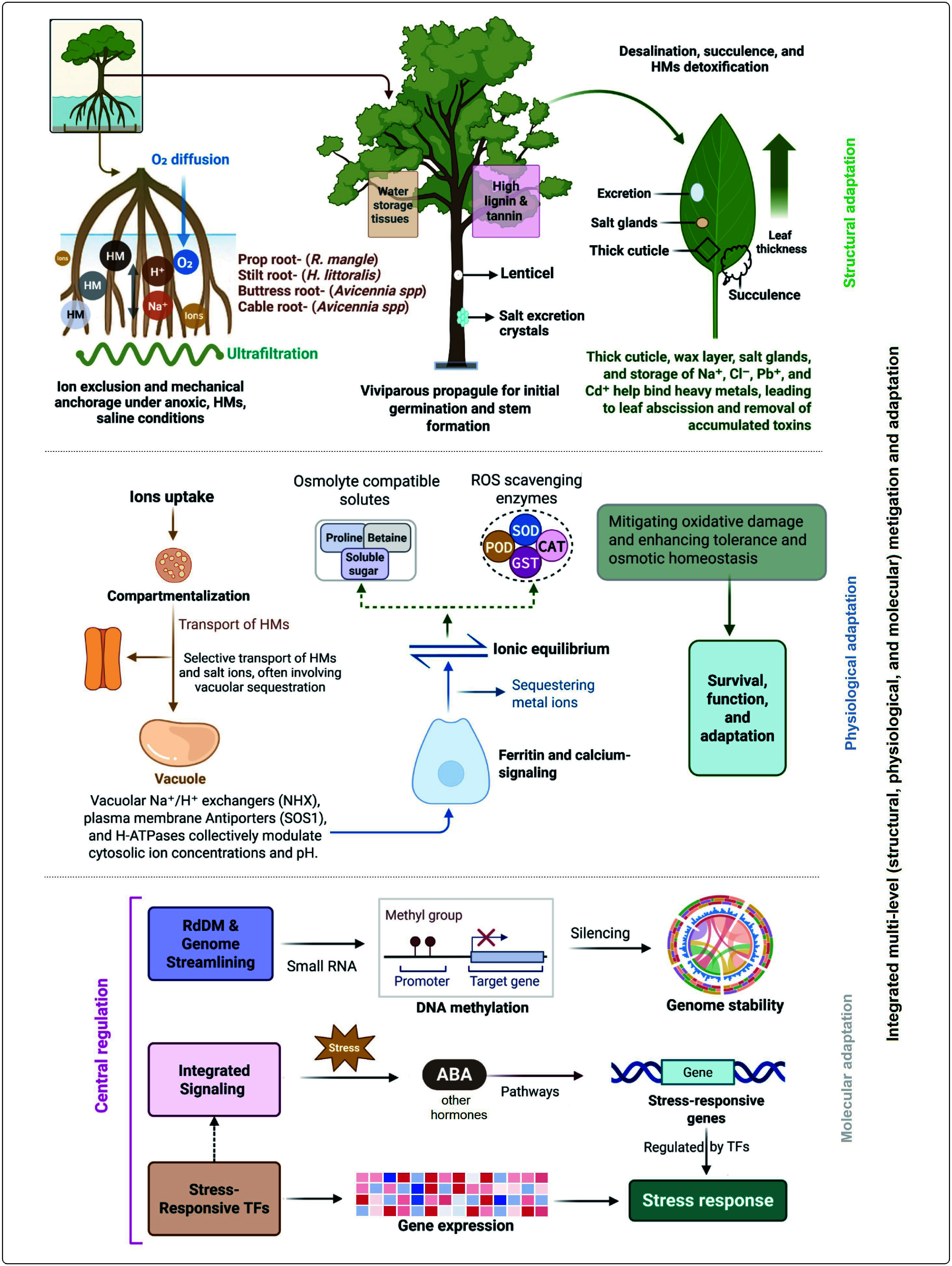
Integration of morphological, physiological, and molecular adaptations of mangrove plants to heavy metals (HMs). Distinct root structures and types function in filtration, viviparous propagule development during germination, and gas exchange through lenticels, while salt excretion from stems, thick leaf structures, leaf-based excretion, waxy layers, and thick cuticles contribute to HM transformation and exclusion. In parallel, coordinated mechanisms of redox balance, ion transport, and osmotic adjustment mitigate HM toxicity. At the molecular level, genome streamlining, integrated signaling pathways, and the regulation of stress-related genes collectively enhance stress tolerance. Together, these mechanisms enable mangrove plants to survive, adapt to, and transform heavy metals within their environment. SOD, superoxide dismutase; POD, peroxidase; CAT, catalase; ROS, reactive oxygen species; RdDM, RNA-directed DNA methylation; ABA, abscisic acid; GST, glutathione S-transferase; TFs, transcription factors.

Mangroves employ precisely coordinated gene networks and epigenetic mechanisms at the molecular level, supporting their stress resilience and adaptive plasticity. High-throughput sequencing in species such as *S. alba* has revealed numerous stress-responsive genes involved in osmolyte biosynthesis, antioxidative defense, ion transport, and hormonal signaling^[225]^. Hormone signaling pathways, particularly abscisic acid-dependent cascades, orchestrate stress responses through stress response genes regulated by central stress-response transcriptional factors in species such as *K. obovata*^[226,227]^. Epigenetic regulation via RNA-directed DNA methylation (RdDM) contributes to genomic stability by silencing transposable elements (TEs). Mangrove genomes may lower TE loads and condense genome sizes, indicating adaptive genome streamlining under environmental stress^[228]^. These integrated multi-level defenses, including structural, physiological, and molecular adaptations, constitute the basis of mangrove resilience to HMs, salinity, and anoxic conditions (Fig. 4). Understanding these strategies not only deepens our insight into the evolution of stress-tolerant plants but also provides a valuable framework for developing resilient crop species under changing climatic conditions.

## Integration of tidal–rhizosphere–plant in HMs transformation

The interplay among tidal dynamics, rhizosphere processes, and plant detoxification mechanisms forms a highly integrated system that controls the ultimate fate of HMs in mangrove ecosystems. At the intertidal scale, sediments are subjected to continuous tidal oscillations that regulate metal distribution. Periodic inundation and exposure cycles alter redox conditions, which in turn influence metal speciation, solubility, and bioavailability. High organic matter content and intense microbial activity within mangrove sediments further enhance the sorption and stabilization of HMs, thereby reducing their mobility. Through these physical and biogeochemical mechanisms, tidal dynamics act as structural and chemical barriers that control metal availability, distribution, and eventual sequestration. Transitioning from the hydrodynamic domain to the biogeochemical environment, the rhizosphere serves as a critical interface mediating HM mobility and transformation. This zone not only traps and stabilizes suspended particulates but also establishes localized chemical gradients that profoundly modify redox potential, pH, and microbial community composition. Root exudates, such as OA, sugars, and phenolic compounds, alter sediment chemistry, chelate metal ions, and influence HM bioavailability, thereby enhancing subsequent detoxification via plant physiological pathways. Elevated enzymatic activities within the rhizosphere simultaneously drive metal detoxification and nutrient cycling, creating reciprocal feedback that sustains system functionality. The synergistic interactions among roots, sediments, and microorganisms form an integrated rhizoremediation network in which biological and geochemical processes converge to transform and immobilize metal contaminants. As a dynamic biogeochemical reactor, the rhizosphere serves as a vital barrier linking abiotic sedimentary processes with plant uptake mechanisms.

**Figure 5:**
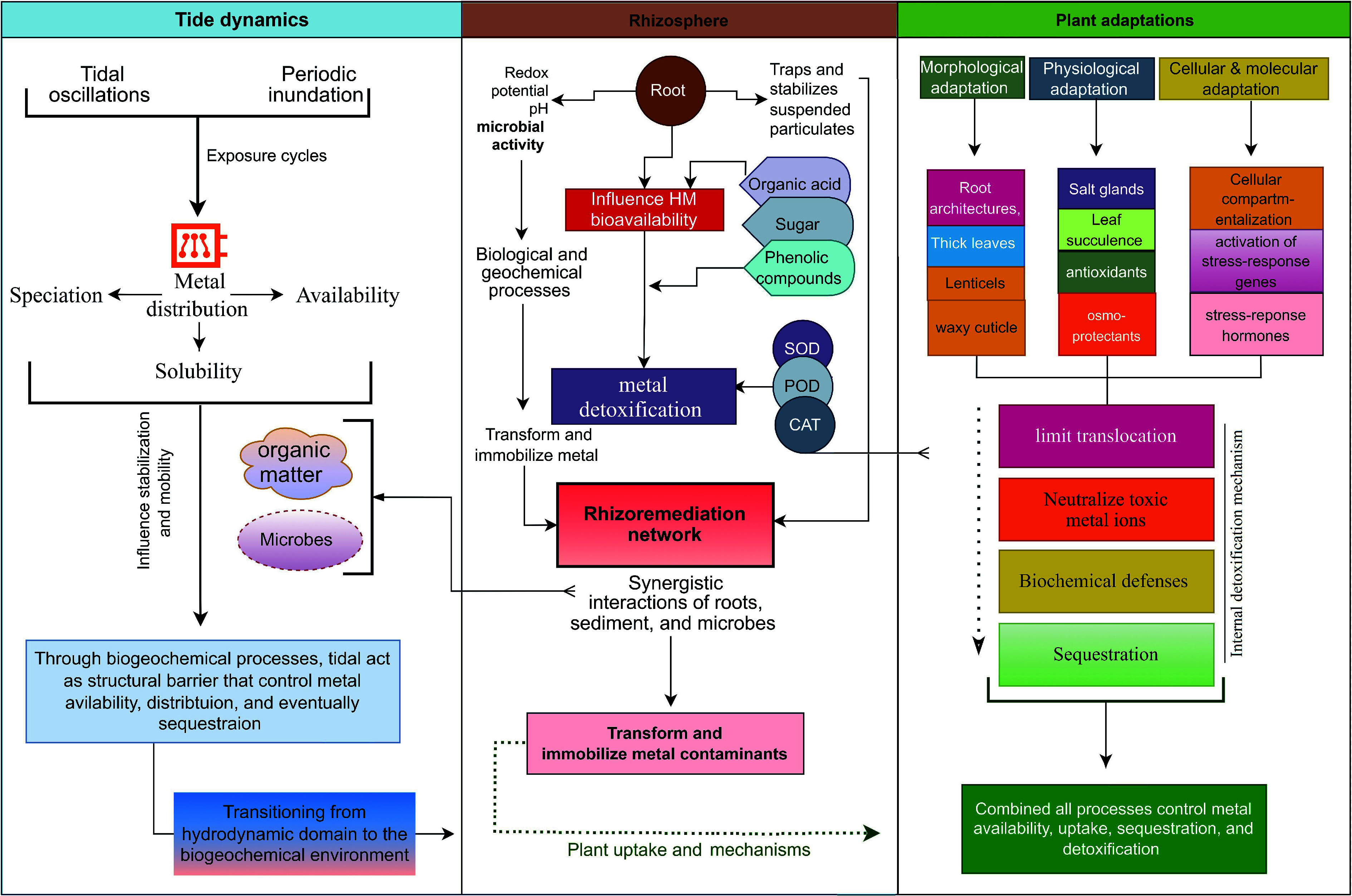
Integrated tide–rhizosphere–plant dynamics in heavy metals (HMs) transformation. Tidal oscillations and periodic inundation regulate HM distribution by influencing metal speciation, solubility, and bioavailability. Tide levels serve as structural barriers, while the organic acids released by mangrove roots and rhizospheric microbial activity further influence HM toxicity and transformation. Biochemical processes involving sugars, phenolic compounds, and antioxidants (SOD, superoxide dismutase; POD, peroxidase; CAT, catalase) contribute to HM immobilization and transformation through rhizoremediation pathways. The synergistic interactions among sediments, roots, and microbes facilitate HM immobilization, uptake, and detoxification via cellular, biochemical, and molecular mechanisms, including ion translocation and intracellular sequestration. Collectively, these interconnected processes control HM transformations from sediments through tide–root–plant interactions within the mangrove ecosystem.

Mangrove plants themselves showed better adaptive and detoxification strategies that ensure survival under conditions of high metal exposure and environmental stress. Morphological adaptations such as complex root architectures, pneumatophores, lenticels, salt glands, and leaf succulence, combined with biochemical and molecular defenses, regulate metal availability, uptake, and sequestration. Externally, extensive root systems and thick leaves limit translocation, while internally, sophisticated biochemical defenses detoxify absorbed metals through chelating agents such as phytochelatins, metallothioneins, and glutathione. These compounds bind and neutralize toxic metal ions, while antioxidant enzymes (e.g., SOD, CAT, and POD), scavenge ROS generated under HM stress. Osmoprotectants such as proline and soluble sugars further support detoxification pathways and maintain cellular integrity and osmotic balance by facilitating metal compartmentalization and stabilization of cellular structures. However, the degree of HM tolerance and detoxification capacity varies among mangrove species and is influenced by their origin, ecological niche, and tidal zonation. For instance, *B. sexangula* has a higher HM accumulation capacity than *S. apetala* because of its native status and better adaptation to local tidal conditions. Similarly, A. marina has greater HM accumulation capacity and tolerance than *K. obovata* and *B. gymnorhiza* due to their stronger physiological and molecular defense mechanisms. Integrating across these hierarchical scales—tidal, rhizosphere, and plant—the transformation of heavy metals operates as a tiered mechanism in which tidal processes regulate external availability, roots function as selective biogeochemical filters, and intracellular detoxification ultimately completes metal exclusion (Fig. 5).

## Conclusions and future outlook

Mangrove ecosystems serve as critical biogeochemical buffers that mitigate HMs pollution in coastal zones. Yet their stability is increasingly threatened by anthropogenic pressures, climate change, expanding tourism, industrial discharges, and biological invasions. Despite these stresses, mangrove employs a suite of adaptive and detoxification mechanisms that collectively regulate metal mobility and toxicity. Tidal hydrodynamics drive redox cycles that control HM speciation and bioavailability, while the rhizosphere, rich in microbial and biochemical activity, mediates metal transformation through root oxygen release, organic exudation, and microbe–root interactions. At the organismal level, selective uptake, root barriers, iron plaque formation, and intracellular chelation help maintain metal homeostasis and protect tissues from oxidative damage. Together, these tidal, rhizosphere, and physiological processes enable mangroves to function as self-sustaining filters that immobilize and transform HMs. However, substantial knowledge gaps remain, including the epigenetics and multi-omics regulation of HM responses, the roles of microbial consortia, and the ecological consequences of competition between native and exotic species, particularly relevant to the region of Hainan, China. As global change accelerates, whether mangrove can maintain their resilience and biogeochemical capacity is a pressing question for coastal management. Strengthening mangrove-based remediation will require prioritizing native, metal-tolerant species, along with long-term monitoring using remote sensing, GIS, and in situ geochemical assessments to detect early signs of metal stress and safeguard ecosystem health.

## Data Availability

All data generated or analyzed during this study are included in this published article.
